# Dimethylarginine Dimethylaminohydrolase/Nitric Oxide Synthase Pathway in Liver and Kidney: Protective Effect of Cyanidin 3-*O*-β-D-Glucoside on Ochratoxin-A Toxicity

**DOI:** 10.3390/toxins4050353

**Published:** 2012-05-08

**Authors:** Valeria Sorrenti, Claudia Di Giacomo, Rosaria Acquaviva, Matteo Bognanno, Ester Grilli, Nicolantonio D’Orazio, Fabio Galvano

**Affiliations:** 1 Department of Drug Sciences, Section of Biochemistry, University of Catania, Italy; Email: sorrenti@unict.it (V.S.); cdigiaco@unict.it (C.D.G.); racquavi@unict.it (R.A.); 2 Department of Agro-forestry, Environmental Science and Technology (STAFA), Mediterranean University of Reggio Calabria, Italy; Email: matteo.bognanno@unirc.it; 3 Department of Morphophysiology and Animal Production (DIMORFIPA), University of Bologna, Italy; Email: ester.grilli@unibo.it; 4 Department of Biochemistry, Section of Human Nutrition, “G. D’Annunzio” University of Chieti, Italy; Email: ndorazio@unich.it

**Keywords:** cyanidin-3-*O*-β-glucoside, DDAH, kidney, NOS, ochratoxin-A

## Abstract

The aim of the present study was to evaluate the effect of long-term cyanidin 3-*O*-β-D-glucoside (C3G) and/or Ochratoxin A (OTA)-exposure on dimethylarginine dimethylamino hydrolase/nitric oxide synthase (DDAH/NOS) pathway in rats. The experiments were performed in rats supplemented with C3G (1 g/kg feed), OTA (200 ppb), and OTA + C3G. After 4 weeks of daily treatment, liver and kidneys were processed for eNOS, iNOS and DDAH-1 Western blotting, nitrite levels evaluation and DDAH activity determination. Results show that OTA is able to induce iNOS both in kidney and liver, whereas OTA is able to induce eNOS and DDAH-1 overexpression and DDAH activation only in kidney, resulting in increased nitrite levels. In kidney of OTA + C3G fed rats, iNOS, eNOS and DDAH-1 expression were less pronounced compared with those observed in the OTA-treated group. Coherent with the decreased iNOS, eNOS and DDAH-1 expression a decrease in nitrite levels and DDAH activity was observed in the OTA + C3G group. Results demonstrate that C3G is able to counteract the deleterious effects of chronic consumption of OTA and also suggest a possible involvement of iNOS-eNOS-DDAH impairment in OTA nephrocarcinogenity.

## 1. Introduction

Ochratoxin A (OTA), or (*R*)-*N*-[(5-chloro-3,4-dihydro-8-hydroxy-3-methyl-1-oxo-1*H*-2-benzopyran-7-yl)carbonyl)-L-phenylalanine, is a natural contaminant of the food chain worldwide [1,2] produced, as a secondary metabolite by some filamentous fungi species belonging to the genera *Aspergillus* and *Penicillium* [3]. It has been reported that OTA is toxic in rodents and other animals [4] and is involved in the development of different type of cancers in rats, mice and humans [5]. However, the mechanism of OTA carcinogenicity has not been definitively elucidated. OTA toxicity and carcinogenity might be attributed to the formation of reactive oxygen species such as the superoxide anion (O_2_–), hydroxyl radical (–OH) and peroxide (ROO–), which induce a wide range of lesions to cell components, or to reduction in antioxidant defenses [6,7]. Moreover, Cavin *et al.* [8] reported an OTA-mediated increase of the inducible nitric oxide synthase (iNOS) expression in a normal rat kidney cell line and in rat hepatocyte cultures, suggesting the induction of both oxidative and nitrosative stress. Strong evidence suggests that nitric oxide (NO), produced by three isoforms of nitric oxide synthases (neuronal NOS, endothelial NOS and inducible NOS), mediates a variety of actions such as vasodilatation, neurotransmission, host defense against bacteria and angiogenesis [9,10].

Although conflicting data has been reported, an overwhelming amount of clinical and experimental evidence suggests a positive association between iNOS/eNOS overexpression, NO production and tumor progression [11,12,13,14]. In particular, NO produced by eNOS may be involved in tumor angiogenesis [15]. Modulation of NO production may therefore play an important role in regulation of angiogenesis and consequently in tumor progression. Kostorou *et al.* reported the involvement of dimethylarginine dimethylaminohydrolase (DDAH) in cerebral tumor growth and development of tumor vasculature [16]; this enzyme metabolizes the endogenous NOS inhibitor asymmetric dimethylarginine (ADMA). Two isoforms of DDAH with distinct tissue distribution have been identified: DDAH-1 and DDAH-2 [17]. Both isoforms have been identified in the kidney and liver tissues, but the expression of the DDAH-1 isoform appears more abundant [18,19]. In consideration of OTA nephrotoxicity and its possible involvement in the development of urinary tract tumors and also in view of the involvement of DDAH and NOS in tumor growth and development of tumor vasculature, the aim of the present study was to evaluate the effect of chronic OTA-exposure on the DDAH/NOS pathway in rats. Moreover, in our previous studies we demonstrated that anthocyanin, cyanidin 3-*O*-β-D-glucoside (C3G), is able to protect human fibroblasts against OTA induced DNA damage *in vitro* [20] and efficiently counteracted deleterious effects of OTA *in vivo* because of its antioxidant and HO-1-inducing properties [7]. Therefore, the present *in vivo* study was performed to evaluate the effect of prolonged C3G and/or OTA-exposure on DDAH/NOS pathway in rats.

## 2. Material and Methods

### 2.1. Chemicals

OTA from *Aspergillus ochraceus* was purchased from Sigma-Aldrich (St Louis, MO, USA) and C3G was purchased from Polyphenols Laboratories (Sandnes, Norway). Mouse monoclonal eNOS antibody was from Sigma Aldrich, rabbit polyclonal iNOS antibody was from SantaCruz Biotechnology (Santa Cruz, CA, USA), goat polyclonal DDAH-1 antibody was from Calbiochem EMD Biosciences, Inc., an affiliate of Merck KGaA (Darmstadt, Germany), anti-actin antibody was from Sigma Aldrich and secondary horseradish peroxidase conjugated anti-mouse, anti-rabbit and anti-goat antibodies were from Amersham Biosciences Piscataway-NJ-USA. The enhanced chemiluminescence system for developing immunoblots and nitrocellulose membranes was purchased from Amersham Pharmacia Biotech (Milan, Italy). All other chemicals were purchased from Merck (Frankfurt, Germany).

### 2.2. Animals and Treatment

The experiments reported in the present paper complied with current Italian and European law (National law: Art 7 D.L. 116 27/01/1992; European law: Directive 2010/63/EU) and met the guidelines of the Institutional Animal Care and Use Committee of University of Catania (Italy). The experiments were performed in male Sprague-Dawley albino rats (150 g body weight and age 45 d at the beginning of experiments). They had free access to water and were kept at room temperature with a natural photo-period (12 h light–12 h dark cycle). Rats were subdivided into four groups (each group consisted of ten animals; each animal was placed into a separate cage) and received the test compounds orally, via their food pellets (20 g feed per rat and day), for 4 weeks. A control group received a commercial balanced standard diet, group C3G received the standard control diet supplemented with C3G (1 g/kg feed), group OTA received the standard control diet supplemented with OTA (200 ppb), group OTA + C3G received the standard diet of the OTA group supplemented with C3G. Both OTA and C3G dosage were chosen according to overall literature data relating to toxic chronic effect and antioxidant properties, respectively. After 4 weeks of daily treatment, animals underwent euthanasia by an overdose of anaesthetic (ethyl urethane, intraperitoneally) and the liver and kidneys of each rat were rapidly removed in a cold room and immediately frozen (−80 °C). Samples were processed within 1 week of collection.

### 2.3. Western Blot Analysis

Tissues were homogenized in 9 volumes of cold PBS. Samples of homogenate were used to evaluate eNOS, iNOS, and DDAH-1 expressions. Whole kidney and liver homogenates were processed for Western blot analysis and protein levels were visualized by immunoblotting as previously described [21] with antibodies against eNOS, iNOS, DDAH-1.

Briefly, 50 μg protein was separated by sodium dodecyl sulfate-polyacrylamide gel electrophoresis and transferred to a nitrocellulose membrane using a semidry transfer apparatus (Bio-Rad, Hercules, CA). The membranes were incubated with 5% milk in 10 mM Tris-HCl (pH 7.4), 150 mM NaCl, and 0.05% Tween 20 buffer at 4 °C overnight. After washing with 150 mM NaCl and 0.05% Tween 20 buffer, the membranes were incubated with a 1:1000 dilution of specific antibody overnight at 4 °C, with constant shaking. The filters were then washed and subsequently probed with horseradish peroxidase–conjugated donkey anti-rabbit, anti-mouse, anti-goat immunoglobulins G at a dilution of 1:2000. Chemiluminescence detection was performed with the Amersham Enhanced Chemiluminescence detection kit according to the manufacturer’s instructions.

Densitometric analysis was then performed and normalized with relative actin.

### 2.4. NO_2_^−^/NO_3_^−^ Quantification

Nitrite, the stable metabolite of nitric oxide, was measured colorimetrically via Griess reaction. Aliquots of homogenates, employed for Western blotting, were preincubated for 30 min at room temperature with 50 μM nicotinamide adenine dinucleotide phosphate (Sigma-Aldrich, St Louis, MO, USA) and 24 mU nitrate reductase (Roche Diagnostics Gmbh, Mannheim, Germany), then the samples were treated with 0.2 U lactate dehydrogenase (Roche, Mannheim, Germany) and 0.5 μmol sodium pyruvate for 10 min. The coloration was developed by adding Griess reagent (1:1, v/v). Finally, after 10 min at room temperature, absorbance was recorded by 96-well plate microtiter (Thermo Labsystems Multiskan, Milford, MA, USA) at λ = 540 nm. Nitrite levels were determined using a standard curve and expressed as nanomoles of NO_2_**^−^**/NO_3_^−^ per milligram of protein. Protein content was determined by Lowry assay [22].

### 2.5. DDAH Enzyme Activity Assay

Tissues were homogenized in 0.1 M phosphate buffer, pH 6.5, containing 2 mM mercapto-ethanol and protease inhibitor cocktail (Sigma-Aldrich) (1:1000); homogenates were centrifuged at 5000 *g* for 60 min, and supernatants were collected for DDAH enzyme activity assay. DDAH enzyme activity was assayed by determining L-citrulline formation in a 96-well microtiter plate according to Knipp and Vasak [23]. One unit of enzyme activity was defined as the amount of enzyme catalyzing the formation of 1 μmol L-citrulline/min at 37 °C.

### 2.6. Statistical Analyses

The data are presented as means ±SD for 4 experiments in triplicate. Student’s t test was used where appropriate; for all analyses, *p* < 0.05 was considered statistically significant. All statistical analyses were performed using the statistical software package SYSTAT, version 9 (Systat, Evanston, IL, USA).

## 3. Results

During the experiment rats showed neither reduced vitality nor evident symptoms of toxicity. No significant differences were observed with regard to body weight and feed intake (data not shown).

**Figure 1:**
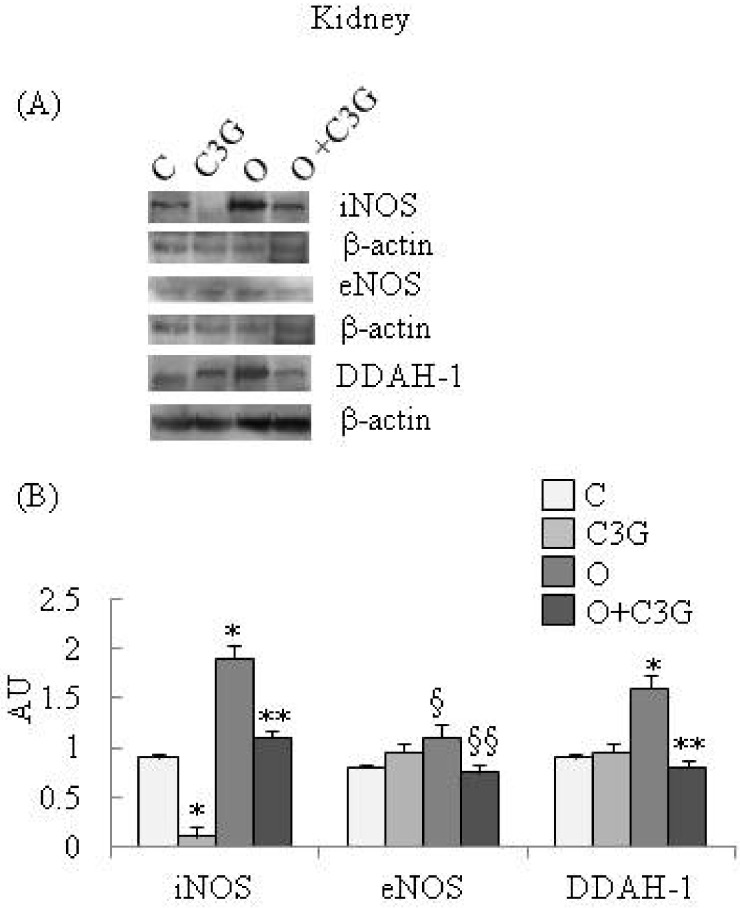
(**A**) Representative Western blotting of iNOS, eNOS and DDAH-1 expression in the kidney; (**B**) Effect of chronic consumption of cyanidin 3-*O*-β-D-glucoside (C3G) or ochratoxin A (O) or O + C3G compared with control treatment (C) on iNOS, eNOS and DDAH-1 protein expression levels in rat kidney. Values are means of 4 experiments in triplicate, with standard deviations represented by vertical bars. * Mean value was significantly different from that of the control group (*p* < 0.005); ** Mean value was significantly different from that of the (O) group (*p* < 0.005); § Mean value was significantly different from that of the control group (*p* < 0.05); §§ Mean value was significantly different from that of the (O) group (*p* < 0.05).

Figure 1A reports representative Western blotting of iNOS, eNOS and DDAH-1 expression in kidney. A significant reduction of iNOS expression is evident in the C3G-fed group whereas an induction of iNOS is evident in the OTA group. The simultaneous intake of both substances resulted in a decrease in iNOS expression with respect to the OTA group. A moderate increase of kidney eNOS expression is evident in the OTA group. The simultaneous intake of both C3G and OTA resulted in a decreased kidney eNOS expression with respect to the OTA group. A significant increase of kidney DDAH-1 expression is evident in OTA groups, whereas C3G, in the C3G + OTA group was able to reduce DDAH-1 induction by OTA (Figure 1B).

**Figure 2:**
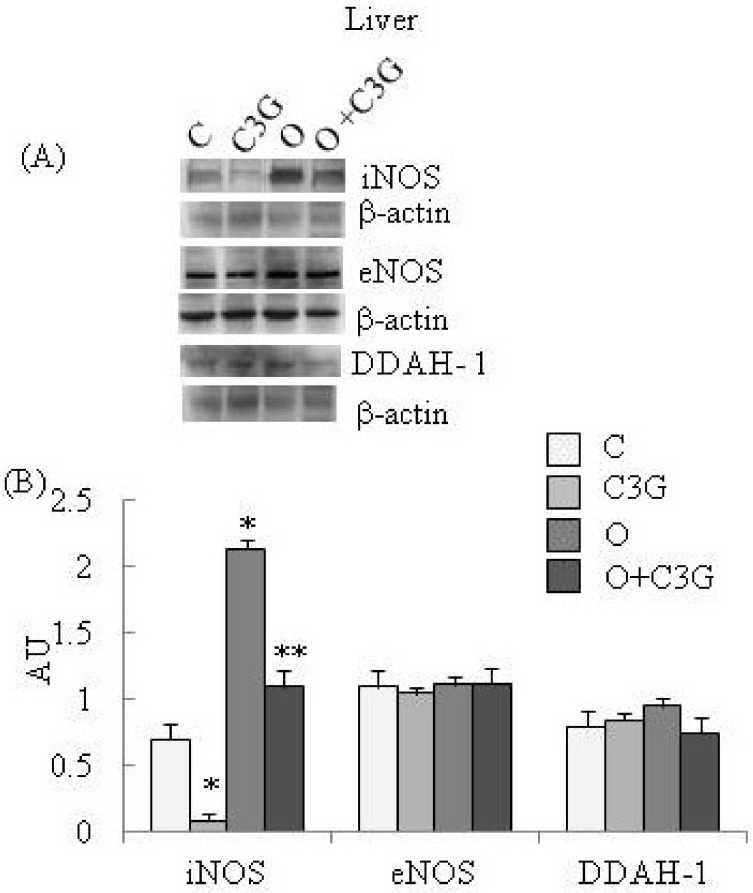
(**A**) Representative Western blotting of iNOS, eNOS and DDAH-1 expression levels in the liver; (**B**) Effect of chronic consumption of cyanidin 3-*O*-β-D-glucoside (C3G) or ochratoxin A (O) or O + C3G compared with control treatment (C) on iNOS, eNOS and DDAH-1 protein expressions in rat liver. Values are means of 4 experiments in triplicate, with standard deviations represented by vertical bars. * Mean value was significantly different from that of the control group (*p* < 0.005); ** Mean value was significantly different from that of the (O) group (*p* < 0.005).

Figure 2A reports representative Western blotting of iNOS, eNOS and DDAH-1 expressions in liver. A significant reduction of iNOS expression is evident in the C3G-treated group whereas an induction of iNOS is evident in the OTA group. The simultaneous intake of both substances resulted in a decreased iNOS expression with respect to the OTA group. None of the dietary treatments significantly affected liver eNOS expression. A moderate increase of liver DDAH-1 expression is evident in OTA groups, whereas C3G, in the C3G + OTA group, was able to reduce DDAH-1 induction by OTA (Figure 2B).

Table 1 reports results regarding kidney DDAH activity and nitrite and nitrate levels. According to DDAH-1 expression a significant increase of DDAH activity is evident in kidney-OTA group, whereas the simultaneous intake of both C3G and OTA resulted in a decrease of kidney DDAH-1 activity with respect to the OTA group. According to iNOS and eNOS expression, nitrite and nitrate levels were decreased in thhe kidney C3G group and increased in kidney OTA group. The simultaneous intake of both C3G and OTA caused a decrease of kidney nitrite and nitrate levels with respect to the OTA group.

**Table 1:** Effect of chronic consumption of ochratoxin A (O), cyanidin 3-*O*-β-D-glucoside (C3G) or O + C3G compared with control treatment (C) on DDAH activity (nmoles citrulline/min/mg prot) and nitrite/nitrate levels (nmoles NO_2_^−^NO_3_^−^/mg prot) in rat kidney. Values are means of 4 experiments in triplicate ± standard deviation. * Mean value was significantly different from that of the control group (*p* < 0.005); ** Mean value was significantly different from that of the (O) group (*p* < 0.005).

Kikney	C	C3G	O	O + C3G
DDAH activity	3.59 ± 0.03	3.58 ± 0.05	5.31 ± 0.04 *	2.93 ± 0.01 **
nitrite/nitrate levels	5.15 ± 0.04	2.01 ± 0.02 *	9.37 ± 0.09 *	6.34 ± 0.08 **

Table 2 reports results regarding liver DDAH activity and nitrite/nitrate levels. None of the dietary treatments significantly affected liver DDAH activity. According to iNOS expression, nitrite and nitrate levels were decreased in liver C3G group and increased in liver OTA group. The simultaneous intake of both C3G and OTA caused a decrease of liver nitrite and nitrate levels with respect to the OTA group.

**Table 2:** Effect of chronic consumption of ochratoxin A (O), cyanidin 3-*O*-β-D-glucoside (C3G) or O + C3G compared with control treatment (C) on DDAH activity (nmoles citrulline/min/mg prot) and nitrite/nitrate levels (nmoles NO_2_^−^NO_3_^−^/mg prot) in rat liver. Values are means of 4 experiments in triplicate ± standard deviation. * Mean value was significantly different from that of the control group (*p* < 0.005); ** Mean value was significantly different from that of the (O) group (*p* < 0.005).

Liver	C	C3G	O	O + C3G
DDAH activity	1.74 ± 0.01	1.58 ± 0.03	1.50 ± 0.01	1.41 ± 0.02
nitrite/nitrate levels	6.86 ± 0.09	3.14 ± 0.02 *	11.42 ± 0.10 *	7.10 ± 0.07 **

## 5. Discussion

In recent years, increasing attention has been focused on the study of the biological effects of phytochemicals in diverse plants [24,25,26], and the number of molecules isolated and characterized continues to increase. Anthocyanins are one kind of phytochemical and they are widely found in plants that are used for food. Glycosides of aglycon cyanidin represent the most abundant anthocyanins in vegetables. Among anthocyanins, C3G, contained in pigmented oranges and fruits of the berry family, has been found to possess several biological properties; it scavenges free radicals [27], suppresses inflammation [28], decreases myocardium damage [29] and protects against endothelial dysfunction [30]. C3G might also help prevent cancer as other anthocyanins do [31].

The present study aimed to verify the hypothesis that prolonged consumption of C3G might counteract OTA-mediated injury. OTA is a widespread mycotoxin, a natural contaminant of moldy food with important implications for animal and human health. Exposure to OTA is a worldwide phenomenon, as evidenced by its detection in sera from human individuals of many countries. Due to a lack of data, the actual health significance of low levels of OTA exposure in humans cannot be evaluated based on epidemiology. Instead, risk assessment has to rely on studies conducted in laboratory animals where it causes various toxic effects, the most relevant being nephrotoxicity and nephrocarcinogenicity in rats [32]. A number of mechanisms have been proposed to account for OTA toxicity and OTA-induced renal tumor formation [8,20,33,34,35].

Several authors and expert groups have concluded that OTA is genotoxic [20,34,36]. However other authors indicate that OTA is unlikely to act through a direct genotoxic mechanism [32,37] and that its carcinogenicity is due to an indirect mechanism such as induction of oxidative stress [33,38]. Other authors hypothesized that OTA may stimulate the production of NO resulting in nitrosative stress and DNA damage [8]. Thus, further research is necessary to describe precisely the molecular mechanisms involved.

The kidney is the target organ of OTA toxicity, probably because OTA is actively accumulated in kidney cells [39]. Nevertheless, OTA has been shown to affect other organs as well, including the liver. OTA has been shown to be hepatotoxic in rats [40]. Although the liver is not the main target organ for OTA, hepatocytes are exposed to OTA since it has to pass through the liver after intestinal absorption [41]. Results obtained in the present study, in line with *in vitro* studies by Cavin [8] and Ferrante [42], allow us to suggest that, through iNOS induction, OTA is able to induce overproduction of NO, both in kidney and liver, resulting in increased nitrite and nitrate levels. Under normal conditions, NO presents a broad range of biological activities; conversely, in excess, it may behave as a toxic radical. In fact, NO is known to react with O_2_– to form the prooxidant peroxynitrite ONOO– [43] with consequent nitrosative stress. As reported in our previous research [7], 4 week-OTA exposure is able to induce oxidative damage, both in kidney and in liver; then overproduction of OTA-induced NO, in the same experimental conditions, may form the prooxidant ONOO, both in kidney and liver. However, our data demonstrate that, only in kidney, OTA is also able to induce eNOS and DDAH-1 overexpression and DDAH activation with further increase of NO levels. These data allow us to speculate that, even if 4 weeks of exposure to OTA were much too low to induce renal tumors, one of the many possible mechanisms by which long-term OTA exposure may cause nephrocarcinogenity might consist in eNOS-DDAH involvement. Therefore, modulation of NO production may play an important role in regulation of angiogenesis and consequently in tumor progression.

According to Wang *et al*. [44], a decreased expression of iNOS was observed in liver and kidney of C3G-supplemented rats. The results of the OTA + C3G group reported here demonstrate that C3G, besides its well-known antioxidant activity, may also act with different molecular mechanisms. In fact, in kidney of rats treated with OTA + C3G, iNOS, eNOS and DDAH-1 expression levels were less pronounced compared with those observed in the OTA group. Coherent with decreased iNOS, eNOS and DDAH-1 expression, a decrease of nitrite and nitrate levels and of DDAH activity was observed in the OTA + C3G group. These results allow us to speculate that long-term consumption of C3G might contribute to reduce OTA-induced tumor growth and tumor angiogenesis in kidney.

Overall, the results obtained in the present study confirm that kidney is the main target organ affected by toxic effects of OTA, but also demonstrate that OTA toxicity to other organs, as well as liver, should not be underestimated. Moreover, the results obtained demonstrate that C3G is able to counteract the deleterious effects of chronic consumption of OTA and confirm the potential effectiveness of dietary strategies to counteract OTA toxicity.
